# Supplementary material for: MetaRibo-Seq measures translation in microbiomes
Source: Nat Commun. 2020 Jun 29;11:3268. doi: 10.1038/s41467-020-17081-z (PMC7324362; doi:10.1038/s41467-020-17081-z)
Supplement: Supplementary file 10 — Supplementary Data 7 [file 41467_2020_17081_MOESM10_ESM.zip › File2/Confidence_High_Taxonomy/415483_out.krona.html]

Javascript must be enabled to view this page.

members
magnitude
magnitudeUnassigned
count
unassigned
taxon
rank

415483\_out

31

31
superkingdom
2

phylum
1
976

200643
class
1

171549
1
order

family
1
815

1
genus
816


SRS015854\_contig\_number\_290
585544
1
species

phylum
30
1239

class
30
186801

186802
order
30

29
family
31979

29
genus
1485

species
2
2293015

SRS042966\_contig\_number\_14865SRS147977\_contig\_number\_12832

25
species

SRS011134\_contig\_number\_46895SRS014855\_contig\_number\_2238SRS015065\_contig\_number\_37942SRS015431\_contig\_number\_78231SRS015578\_contig\_number\_30852SRS015579\_contig\_number\_20735SRS016095\_contig\_number\_contig-100\_1350.141630SRS018623\_contig\_number\_20831SRS019068\_contig\_number\_2215SRS022609\_contig\_number\_contig-100\_2289.234160SRS049773\_contig\_number\_36879SRS051031\_contig\_number\_650SRS063985\_contig\_number\_13088SRS075821\_contig\_number\_17524SRS076929\_contig\_number\_22030SRS077552\_contig\_number\_26998SRS1041038\_contig\_number\_14827SRS1055022\_contig\_number\_18870SRS142503\_contig\_number\_44191SRS143466\_contig\_number\_contig-100\_835.178786SRS143523\_contig\_number\_20920SRS144135\_contig\_number\_4607SRS148159\_contig\_number\_21808SRS148424\_contig\_number\_24527SRS149181\_contig\_number\_20960
1262828

2
species

SRS097889\_contig\_number\_41363SRS143780\_contig\_number\_32451
1896990

1
family
186806

genus
1
1730

1
species

SRS015663\_contig\_number\_23001
39496
